# Postoperative high-density lipoprotein cholesterol level: an independent prognostic factor for gastric cancer

**DOI:** 10.3389/fonc.2022.884371

**Published:** 2022-07-18

**Authors:** Chenxi Li, Yan Fu, Qiuwen Li, Xuhui Yang, Wenying Wang, Xin Jin, Lihua Bian, Hui Zhao, Donghui Li, Jie Gao, Nan Du, Liang Peng

**Affiliations:** ^1^ Senior Department of Oncology, The Fifth Medical Center of People’s Liberation Army (PLA) General Hospital, Beijing, China; ^2^ Senior Department of Obstetrics and Gynecology, The Seventh Medical Center of People' s Liberation Army General Hospital, Beijing, China; ^3^ Senior Department of Hepato-Pancreato-Biliary Surgery, The First Medical Center of People' s Liberation Army General Hospital, Beijing, China; ^4^ Department of Obstetrics and Gynecology, Hainan Hospital of People' s Liberation Army General Hospital, Sanya, China

**Keywords:** preoperative serum lipids, postoperative serum lipids, gastric cancer, prognosis, overall survival, disease-free survival

## Abstract

**Objective:**

The relationship between serum lipids and prognosis of gastric cancer has not been confirmed. Our purpose in the study was to investigate the associations between preoperative and postoperative serum lipids level and prognosis in patients with gastric cancer.

**Methods:**

A retrospective study was performed on 431 patients who received radical (R0) gastrectomy from 2011 to 2013. Preoperative and postoperative serum lipids level were recorded. Clinical-pathological characteristics, oncologic outcomes, disease-free survival (DFS) and overall survival (OS) were collected. The prognostic significance was determined by Kaplan-Meier analysis and Cox proportional hazards regression model.

**Results:**

There was no significant difference in DFS and OS according to preoperative serum lipids level. Regarding postoperative serum lipids level, compared to normal high-density lipoprotein cholesterol (HDL-C), low postoperative HDL-C level indicated a shorter OS (hazard ratio: 1.76, 99% confidence interval: 1.31–2.38; P=0.000) and a shorter DFS (hazard ratio: 2.06, 99% confidence interval: 1.55–2.73; P=0.000). However, other postoperative serum lipid molecules were not associated with DFS and OS.

**Conclusion:**

Postoperative HDL-C might be an independent prognostic factor of gastric cancer.

## Introduction

Gastric cancer is the fifth most diagnosed malignancy worldwide (1) and ranks as the third most common cause of cancer related deaths worldwide (2). Although the popularization of early cancer screening has significantly improved the diagnosis rate of early gastric cancer, GC is still frequently advanced stage at diagnosis. With the progress of medicine, the treatment of GC has been improved, but the prognosis of GC is still poor, and the five-year survival rate is about 53% (3). In addition to the stage of cancer at presentation, many other factors of the patient will also affect the prognosis of gastric cancer. Thus, it is of great significance to explore new potential predictors of long-term prognosis. Serum lipid components, including total cholesterol (TC), triglycerides (TG), high-density lipoprotein cholesterol (HDL-C), low-density lipoprotein cholesterol (LDL-C), and apo lipoprotein. So far, the influence of serum lipids on cancer is unclear. It has been reported that preoperative lipid level is associated with prognosis of non-metastatic colorectal cancer (4). Currently, researchers have found that TC may play an important role in the development of gastric cancer (5); low HDL-C and high LDL-C levels may increase the risk of GC, but no lipid components was associated with OS of GC (6). One study reported that preoperative serum ApoB/ApoA1 ratio as a novel prognostic indicator of GC, and no association of lipid markers with gastric cancer was shown (7). However, another study found a different conclusion, which suggested that HDL-C is closely related to the prognosis of gastric cancer (8). Therefore, the impact of serum lipids level on outcomes after surgery is less well confirmed, especially few studies have focused on the relationships of postoperative serum lipid components with the prognosis of gastric cancer. In this study, we aim to comprehensively investigate the relationships between preoperative and postoperative serum lipids and prognosis of gastric cancer.

## Materials and methods

Patients who received gastrectomy for gastric cancer in Chinese PLA General Hospital from January 1, 2011 to December 31, 2013 were included in this study. The study was approved by the research ethics committee of Chinese PLA General Hospital. Preoperative histological confirmation of the tumor was determined by endoscopic biopsy. Subtotal or total gastrectomy was performed according to tumor location, histopathology and the possibility of obtaining negative resection margins. All postoperative patients were followed up according to guidelines of gastric cancer from surgery to January 1, 2021. The date of tumor recurrence and death was recorded. The endpoint was defined as death or last follow-up. The overall survival was based on the period from histological confirmation of GC to the end of follow-up or endpoint, and the disease-free survival was the period from surgery to the gastric cancer recurrence or metastasis. The following clinical information was recorded in detail: the sex and age of the patient; preoperative serum lipids, body mass index (BMI) and fasting blood sugar; BMI and fasting blood sugar of 6 months after operation; operative site; operative procedure; postoperative pathological results; cancer stage (the pTNM classification was updated to the 8th edition); diet intake volume. Patients with metastatic stage IV disease, serious cardiovascular and cerebrovascular diseases, diabetes, serious thyroid disease, oral lipid-lowering drugs, a non-R0 resection, overall survival of less than 6 months and incomplete information were excluded from the analysis.

The serum lipids and fasting blood sugar were measured in early morning samples obtained before breakfast (at least 8 hours of fasting) within 1 week before surgery and 6 months after surgery by a Cobas c 701 chemistry analyzer (Roche). The low and high reference values were 5.7 mmol/L for TC, 1.7 mmol/L for TG, 1.15 mmol/L for HDL-C, and 3.37 mmol/L for LDL-C. Fasting blood sugar is classified as low group (<6.1 mmol/L), normal group (6.1-7.0 mmol/L) and high group (≥7.0 mmol/L). BMI calculate as weight [kg]/height [m^2^]. In this study, patients with BMI less than 18.5 kg/m^2^ were defined as low group, over 25 kg/m^2^ were defined as high group, and others were normal group. The diet intake volume will reduce after surgery of most patients. Considering the preoperative diet intake volume as 100%, the intake volume increased to 75% of patients at 6 months after surgery was defined as normal group and less than 75% was defined as low group.

Statistical analyses were performed with SPSS version 22.0 software. Continuous variables were presented as the mean ± standard deviation (x ± s). Chi-square test was used to compare categorical variables between groups and the results were described as the percentage (%). The OS and DFS after surgery were calculated using Kaplan-Meier’s method. The potential prognostic factors of GC were explored in univariate and multivariable analysis using a Cox proportional hazards regression model. A *P* value < 0.05 was considered statistically significant in all statistical analyses.

## Results

A total of 431 patients were enrolled in this study. The mean duration of follow-up was 107.1 ± 10.8months (range 85.4-133.6months). The general characteristics of the patients are summarized in **Table 1**. Males were the majority in this study, the male to female ratio was 332:99. The mean age was 55 years. In respect to tumor location, 125 cases (29.0%) were in the upper third, 119 cases (27.6%) were in the middle third, 154 cases (35.7%) were in the lower third, and 33 cases (7.7%) were in the diffuse tumor site. Most of the tumors progressed locally and penetrated the serosa (283 cases of T4 tumors, 65.7%). Lymph node metastasis was common (n = 335, 77.7%). There were 34 patients (7.9%) in stage I, 119 (27.6%) in stage II, and 278 (64.5%) in stage III. On pathology, only 21 cases were signet ring cell carcinoma (4.9%), most of the pathological types were adenocarcinoma (255 cases, 59.2%) and mixed type (155 cases, 36.0%). The degree of pathological differentiation is recorded in detail as follows:362 cases (84.0%) were poorly differentiated; only 12cases (2.8%) were well differentiated, and the others were moderately differentiated (n= 57, 13.2%). In addition, 113 (26.2%) of the GC cases had a positive family history of cancer, either in first- or second-degree relatives. Regarding the operative procedure, total resection was performed in 37.4% (n=161) of the patients, distal resection was performed in 42.2% (n=182) of the patients and other patients (n=88, 20.4%) were performed proximal resection. The diet intake volume of most patients (n=356, 82.6%) increased to 75% after 6 months of surgery. Lipid profile, fasting blood sugar and BMI information are summarized in **Table 2**. As regards lipid profile (before surgery), the distributions were as follows: TG <1.7 mmol/L (170, 39.4%) versus TG≥1.7mmol/L (261, 60.6%); TC<5.7mmol/L(318, 73.8%) versus TC≥5.7mmol/L (113, 26.2%); HDL-C <1.15mmol/L (193, 44.8%) versus HDL-C≥1.15mmol/L (238, 55.2%); LDL-C<3.37 mmol/L (295, 68.4%) versus LDL-C ≥3.37mmol/L (136, 31.6%). Six months after operation, the proportions of TC <5.7 mmol/L, TG <1.7 mmol/L, LDL<3.37 mmol/L and low BMI were significantly increased.

**Table 1:** Clinicopathological characteristics in the 431 gastric cancer patients.

Characteristics	Patients	%
Age (year)		
<60	281	65.2
≥60	150	34.8
Sex		
Male	332	77.0
Female	99	23.0
Tumor location		
Lower third	154	35.7
Middle third	119	27.6
Upper third	125	29.0
Diffuse	33	7.7
Differentiation		
Well	12	2.8
Moderate	57	13.2
Poor	362	84.0
T stage		
1	31	7.2
2	47	10.9
3	70	16.2
4	283	65.7
N stage		
0	96	22.3
1	86	20.0
2	102	23.7
3a	92	21.3
3b	55	12.8
TNM stage		
I	34	7.9
II	119	27.6
III	278	64.5
Histological type		
Adenocarcinoma	255	59.2
Signet Ring Cell	21	4.9
Mixed	155	36.0
Family history		
Yes	113	26.2
No	318	73.8
Operative procedure		
Total	161	37.4
Distal	182	42.2
Proximal	88	20.4
Diet intake volume		
Low	75	17.4
Normal	356	82.6

**Table 2:** characteristics of lipid profile, BMI and fasting blood sugar in the 431 gastric cancer patients.

Characteristics	Patients	%
**Preoperative**		
TC (mmol/L)		
<5.7	318	73.8
≥5.7	113	26.2
TG (mmol/L)		
<1.7	170	39.4
≥1.7	261	60.6
HDL (mmol/L)		
<1.15	193	44.8
≥1.15	238	55.2
LDL (mmol/L)		
<3.37	295	68.4
≥3.37	136	31.6
BMI		
low	64	14.8
normal	198	45.9
high	169	39.2
Fasting blood sugar		
<6.1	397	92.1
6.1-7.0	26	6.0
≥7.0	8	1.9
**Postoperative**		
TC (mmol/L)		
<5.7	339	78.7
≥5.7	92	21.3
TG (mmol/L)		
<1.7	272	63.1
≥1.7	159	36.9
HDL (mmol/L)		
<1.15	191	44.3
≥1.15	240	55.7
LDL (mmol/L)		
<3.37	358	83.1
≥3.37	73	16.9
BMI		
low	110	25.5
normal	250	58.0
high	71	16.5
Fasting blood sugar(mmol/L)		
<6.1	403	93.5
6.1-7.0	23	5.3
≥7.0	5	1.2

In this study, the overall median DFS was 52.3 ± 34.4 months. The 3- and 5-year DFS rates were 61.3% and 43.9%, respectively. In a univariate Cox proportional hazards model of GC with DFS (**Table 3**, left panel), the factors associated with the gastric cancer DFS were the sex, T stage, N stage, TNM stage, differentiation, family history, operative procedure, preoperative BMI and postoperative HDL-C levels. To determine which factors may affect the prognosis of gastric cancer, all factors were further subjected to multivariate regression analyses. As shown in **Table 3** (right panel), moderate and poor differentiations of tumor (HR: 3.78, 99% CI: 1.26–11.36, P = 0.002; HR: 3.78, 99% CI: 1.20–11.93, P = 0.003, respectively), T2, T3 and T4 of tumor (HR: 2.22, 99% CI: 0.96–5.18, P = 0.015; HR: 4.78, 99% CI: 2.16–10.57, P = 0.000; HR: 6.30, 99% CI: 2.99–13.30, P = 0.000, respectively). N1, N2, N3a and N3b of tumor (HR: 2.10, 99% CI: 1.35–3.28, P = 0.000; HR: 2.56, 99% CI: 1.69–3.88, P = 0.000; HR: 2.47, 99% CI: 1.62–3.77, P = 0.000, HR: 4.91, 99% CI: 3.00–8.02, P = 0.000, respectively); preoperative fasting blood sugar in 6.1-7.0mmol/(HR:1.82, 99% CI: 0.99-3.34, P = 0.012); postoperative HDL-C<1.15mmol/L (HR: 2.06, 99% CI: 1.55–2.73, P = 0.000) were related with poor DFS.

**Table 3:** Univariate and multivariate cox proportional hazard model of gastric cancer with DFS.

	Univariate analysis		Multivariate analysis	
	HR (99% CI)	*P*-value	HR (99% CI)	*P*-value
Age (year)		0.283		
<60	1			
≥60	1.12 (0.85-1.47)			
Sex		0.006		
Male	1			
Female	0.71 (0.51-0.98)			
Tumor location		0.237		
Upper third	1			
Middle third	0.97 (0.69-1.37)	0.822		
Lower third	0.81 (0.59-1.13)	0.104		
Diffuse	1.12 (0.66-1.92)	0.574		
Differentiation		0.007		0.008
Well	1		1	
Moderate	3.71 (1.27-10.82)	0.002	3.78 (1.26-11.36)	0.002
Poor	3.56 (1.16-10.92)	0.003	3.78 (1.20-11.93)	0.003
T stage		0.000		0.000
1	1		1	
2	1.91 (0.84-4.31)	0.041	2.22 (0.96-5.18)	0.015
3	4.28 (1.99-9.23)	0.000	4.78 (2.16-10.57)	0.000
4	5.41 (2.65-11.03)	0.000	6.30 (2.99-13.30)	0.000
N stage		0.000		0.000
0	1		1	
1	1.72 (1.23-2.62)	0.001	2.10 (1.35-3.28)	0.000
2	2.35 (1.58-3.51)	0.000	2.56 (1.69-3.88)	0.000
3a	2.78 (1.84-4.19)	0.000	2.47 (1.62-3.77)	0.000
3b	5.55 (3.45-8.92)	0.000	4.91 (3.00-8.02)	0.000
TNM stage		0.000		
I +II	1			
III	3.50 (2.60-4.72)			
Histological type		0.805		
Adenocarcinoma	1			
Signet Ring Cell	0.92 (0.49-1.73)	0.734		
Mixed	1.06 (0.80-1.39)	0.618		
Family history		0.008		
Yes	1			
No	1.37 (1.01-1.86)			
Operative procedure		0.011		
Total	1			
Distal	0.71 (0.53-0.96)	0.003		
Proximal	0.88 (0.62-1.26)	0.364		
Diet intake volume		0.880		
Low	1			
Normal	0.98 (0.70-1.38)			
Pre TC (mmol/L)		0.997		
<5.7	1			
≥5.7	1.00 (0.74-1.35)			
Pre TG (mmol/L)		0.882		
<1.7	1			
≥1.7	0.99 (0.75-1.29)			
Pre HDL (mmol/L)		0.489		
<1.15	1.07 (0.83-1.40)			
≥1.15	1			
Pre LDL (mmol/L)		0.100		
<3.37	1			
≥3.37	0.84 (0.63-1.11)			
Pre BMI		0.004		0.021
Low	1		1	
Normal	0.63 (0.42-0.94)	0.003	0.76(0.51-1.14)	0.080
High	0.83 (0.55-1.23)	0.215	1.03(0.68-1.56)	0.848
Pre-fasting blood sugar(mmol/L)		0.956		0.012
<6.1	1		1	
6.1-7.0	1.04 (0.59-1.85)	0.846	1.82(0.99-3.34)	0.012
≥7.0	0.92 (0.37-2.32)	0.822	0.60(0.23-1.54)	0.158
Post TC (mmol/L)		0.649		
<5.7	1			
≥5.7	1.06 (0.77-1.45)			
Post TG (mmol/L)		0.914		
<1.7	1			
≥1.7	1.01 (0.77-1.33)			
Post HDL (mmol/L)		0.000		
<1.15	2.02 (1.55-2.64)	2.06 (1.55-2.73)	0.000
≥1.15	1		1	
Post LDL (mmol/L)		0.985		
<3.37	1			
≥3.37	1.00 (0.71-1.42)			
Post BMI		0.341		
Low	1			
Normal	1.12 (0.81-1.54)	0.379		
High	0.92 (0.61-1.41)	0.623		
Post fasting blood sugar(mmol/L)		0.424		
<6.1	1			
6.1-7.0	1.33 (0.76-2.35)	0.193		
≥7.0	1.09 (0.34-3.47)	0.856		

**Figure 1:**
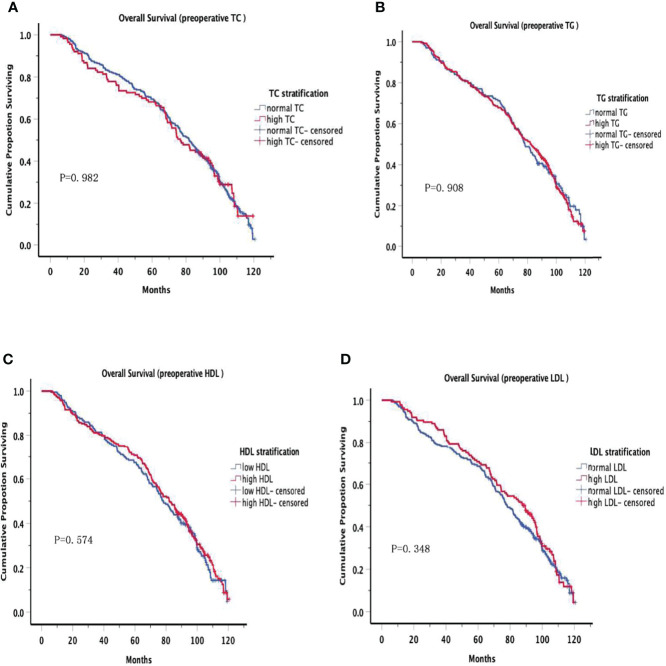
Overall Survival in preoperative serum lipid. Overall Survival of patients grouped by serum lipid levels before surgery, no significant differences were observed at all levels.

**Figure 2:**
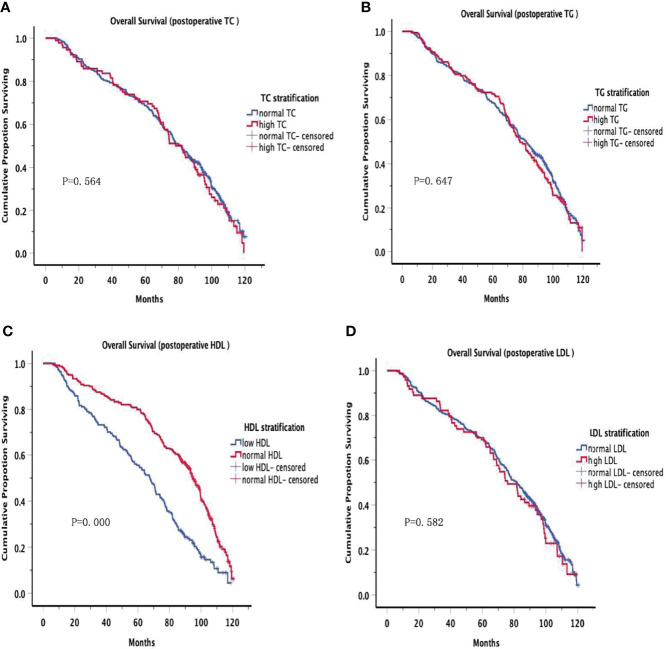
Overall Survival in postoperative serum lipid. There were significant differences in the low HDL and normal HDL groups (P=0.000). However, no significant differences were observed in other groups.

The OS curves are shown in **Figures 1A–D** for preoperative lipids groups; No significant differences were observed in those stratified analyses. **Figure 2** show the postoperative OS curves for the lipids groups. In the Kaplan-Meier curve for HDL-C after surgery, the 5-year OS rate was greater in the normal group than in the low group (80.0% versus 55.5%) (**Figure 2C**), there were significant differences in the two groups (P=0.000). Regarding the TC, TG and LDL-C after operation (**Figures 2A, B, D**), no statistically significant differences were observed in those stratified analyses (P = 0.564, P = 0.647, P = 0.582). Furthermore, Cox proportional hazard model was used to analyze which factors could predict OS of gastric cancer. According to the univariate analysis, the factors related with the gastric cancer overall survival were the sex, tumor location, differentiation, T stage, N stage, TNM stage, family history, operative procedure, preoperative BMI and postoperative HDL-C (**Table 4** left panel). The results of the multivariate analysis of factors influencing the gastric cancer OS are presented in **Table 4** (right panel), increasing T stage, increasing lymph node stage, unnormal preoperative BMI and low postoperative HDL-C all indicated a low mortality rate.

**Table 4:** Univariate and multivariate cox proportional hazard model of gastric cancer with OS.

	Univariate analysis		Multivariate analysis	
	HR (99% CI)	P-value	HR (99% CI)	*P*-value
Age (year)		0.882		
<60	1			
≥60	1.02 (0.75-1.37)			
Sex		0.028		
Male	1			
Female	0.73 (0.51-1.05)			
Tumor location		0.030		
Upper third	1			
Middle third	1.06 (0.72-1.55)	0.694		
Lower third	0.89 (0.62-1.26)	0.376		
Diffuse	1.67 (0.96-2.90)	0.018		
Differentiation		0.048		
Well	1			
Moderate	2.67 (0.91-7.79)	0.019		
Poor	2.93 (0.95-9.07)	0.014		
T stage		0.000		0.000
1	1		1	
2	2.21 (0.82-5.96)	0.040	2.39 (0.88-6.52)	0.025
3	3.09 (1.18-8.10)	0.003	2.83 (1.06-7.58)	0.007
4	5.99 (2.49-14.39)	0.000	6.80 (2.76-16.78)	0.000
N stage		0.000		0.000
0	1		1	
1	2.08 (1.29-3.35)	0.000	2.85 (1.72-4.72)	0.000
2	2.48 (1.57-3.92)	0.000	3.00 (1.86-4.84)	0.000
3a	3.11 (1.95-4.96)	0.000	2.97 (1.84-4.80)	0.000
3b	6.10 (3.64-10.21)	0.000	6.13 (3.59-10.46)	0.000
TNM stage		0.000		
I+ II	1			
III	3.91 (2.77-5.52)			
Histological type		0.434		
Adenocarcinoma	1			
Signet Ring Cell	1.31 (0.68-2.51)	0.288		
Mixed	1.11 (0.82-1.51)	0.363		
Family history		0.049		
Yes	1			
No	1.29 (0.93-1.80)			
Operative procedure		0.003		
Total	1			
Distal	0.66 (0.48-0.91)	0.001		
Proximal	0.75 (0.51-1.10)	0.050		
Diet intake volume		0.498		
Low	1			
Normal	0.91 (0.64-1.30)			
Pre TC (mmol/L)		0.982		
<5.7	1			
≥5.7	1.00 (0.72-1.39)			
Pre TG (mmol/L)		0.908		
<1.7	1			
≥1.7	1.01 (0.76-1.36)			
Pre HDL (mmol/L)		0.574		
<1.15	1.07 (0.80-1.42)			
≥1.15	1			
Pre LDL (mmol/L)		0.348		
<3.37	1			
≥3.37	0.89 (0.66-1.22)			
Pre BMI		0.000		0.001
Low	1		1	
Normal	0.52 (0.34-0.79)	0.000	0.58 (0.38-0.88)	0.001
High	0.62 (0.41-0.95)	0.004	0.79 (0.51-1.22)	0.163
Pre fasting blood sugar(mmol/L)		0.741		
<6.1	1			
6.1-7.0	1.14 (0.63-2.09)	0.570		
≥7.0	1.22 (0.48-3.07)	0.586		
Post TC (mmol/L)		0.565		
<5.7	1			
≥5.7	1.08 (0.77-1.51)			
Post TG (mmol/L)		0.648		
<1.7	1			
≥1.7	1.05 (0.79-1.41)			
Post HDL (mmol/L)		0.000		
<1.15	2.02 (1.51-2.70)	1.76 (1.31-2.38)	0.000
≥1.15	1	1	
Post LDL (mmol/L)		0.582		
<3.37	1			
≥3.37	1.08 (0.74-1.58)			
Post BMI		0.227		
Low	1			
Normal	0.86 (0.61-1.20)	0.242		
High	0.74 (0.46-1.17)	0.092		
Post fasting blood sugar(mmol/L)		0.231		
<6.1	1			
6.1-7.0	1.49 (0.82-2.70)	0.087		
≥7.0	1.00 (0.27-3.67)	0.998		

The association between postoperative HDL-C level and clinicopathological characteristics is summarized in **Table 5**. Of note, HDL-C level was significantly correlated with T stage (P=0.001), lymph node stage (P=0.010), TNM stage (P=0.000) and operative procedure (P=0.007). However, there was no significant relationships between postoperative HDL-C level and age, sex, tumor location, differentiation, histological type, postoperative BMI, postoperative fasting blood sugar, diet intake volume and family history.

**Table 5:** Characteristics of the normal-HDL and low-HDL groups in 431 patients (after surgery).

Characteristics	Normal HDL (%)	Low HDL (%)	*P*-value
Age (year)			0.130
<60	164 (68.3)	117 (61.3)	
≥60	76 (31.7)	74 (38.7)	
Sex			0.176
Male	179 (74.6)	153 (80.1)	
Female	61 (25.4)	38 (19.9)	
Tumor location			0.550
Upper third	71 (29.6)	54 (28.3)	
Middle third	64 (26.7)	55 (28.8)	
Lower third	90 (37.5)	64 (33.5)	
Diffuse	15 (6.3)	18 (9.4)	
Differentiation			0.979
Poor	201 (83.8)	161 (84.3)	
Moderate	32 (13.3)	25 (13.1)	
Well	7 (2.9)	5 (2.6)	
T stage			0.001
1	21 (8.8)	10 (5.2)	
2	38 (15.8)	9 (4.7)	
3	35 (14.6)	35 (18.3)	
4	146 (60.8)	137 (71.7)	
N stage			0.010
0	67 (27.9)	29 (15.2)	
1	43 (17.9)	43 (22.5)	
2	60 (25.0)	42 (22.0)	
3a	45 (18.8)	47 (24.6)	
3b	25 (10.4)	30 (15.7)	
TNM stage			0.000
I+ II	105(43.8)	48 (25.1)	
III	135 (56.3)	143(74.9)	
Histological type			0.619
Adenocarcinoma	145 (60.4)	110 (57.6)	
Signet Ring Cell	13 (5.4)	8 (4.2)	
Mixed	82 (34.2)	73 (38.2)	
Family history			0.987
Yes	63 (26.3)	50 (26.2)	
No	177 (73.8)	141 (73.8)	
Operative procedure			0.007
Total	74 (30.8)	87 (45.5)	
Distal	112 (46.7)	70 (36.6)	
Proximal	54 (22.5)	34 (17.8)	
Diet intake volume			0.180
Low	47 (19.6)	28 (14.7)	
Normal	193 (80.4)	163 (85.3)	
BMI			0.149
Low	59 (24.6)	51 (26.7)	
Normal	134 (55.8)	116 (60.7)	
High	47 (19.6)	24 (12.6)	
Fasting blood sugar(mmol/L)			0.505
<6.1	223 (92.9)	180 (94.2)	
6.1-7.0	15 (6.3)	8 (4.2)	
≥7.0	2 (0.8)	3 (1.6)	

## Discussion

With the improvements in people’s living conditions and the prolongation of average life expectancy, there is an increasing number of people who are suffered from dyslipidemia. It is well known that LDL-C is called “bad” cholesterol, and positively correlated with cardiovascular and cerebrovascular diseases. On the contrary, HDL-C is called “good” cholesterol, and has a protective effect on cardiovascular and cerebrovascular system. So far, the roles played by lipids in cancer prognosis is a controversial area as there are studies reporting positive, negative or no influence of lipids on the advancement of cancer (6–9). Therefore, this study examined the impact of preoperative and postoperative serum lipids level on DFS and OS after resection for gastric.

HDL-C is known as an antioxidant and anti-inflammatory factor, which is one of the types of cholesterol (10). The major function of HDL-C is to maintain normal cell cholesterol homeostasis by removing excess cholesterol from an intracellular pool (11). Many researchers have previously investigated whether there is a relationship between serum HDL-C and tumorigenesis. Patients with gastrointestinal cancer have lower HDL-C level, when compared with normal controls (12). Low level of serum HDL-C may increase risk of gastric cancer (6, 13, 14), the possible reason is that H. pylori infection reduces HDL-C, which is an important risk factor for gastric cancer (15). As for the prognosis, some authors reported that HDL-C did not affect the OS and PFS of gastric cancer (6, 7). However, another study showed that low level of serum HDL-C is one of the factors of poor prognosis in gastric cancer (8). In this study, we found that postoperative HDL-C level no significant change compared to preoperative level. The positive correlation between postoperative HDL-C and T stage, N stage, and TNM stage indicates a more advanced tumor in patients with lower HDL-C level. In our study, the preoperative HDL-C showed negative association with the DFS and OS in gastric cancer. In addition, we also focused on the relationship between postoperative HDL-C level and prognosis of gastric cancer, which has rarely been reported. We found that low postoperative HDL-C level is associated with poor prognosis of gastric cancer. Therefore, the results of our study suggest that postoperative HDL-C level is an independent risk factor for predicting prognosis of gastric cancer.

Some studies have confirmed that high level of LDL-C and low level of HDL-C were risk factors for gastric cancer (6, 16, 17). Higher LDL-C have been reported to relate to pro-inflammatory activity and affect the suppression of the host immune system (18, 19). The results are inconsistent whether TC or TG is risk factor of gastric cancer (14, 20–22). As for the relationship between serum lipids and prognosis of gastric cancer, similar to our study which no association of preoperative TG, TC and LDL-C with gastric cancer was shown (6, 7). However, another study showed LDL-C as an independent prognosis of gastric cancer (23). Similarly, this study investigated the relationship between postoperative LDL-C, TG, TC and prognosis of gastric cancer. Eventually, no correlation between them and prognosis of gastric cancer was be found.

To the best of our knowledge, few studies that focus on the prognostic role of the postoperative lipids level in patients with gastric cancer. Thus, in our research, we incorporated both preoperative and postoperative TG, TC, LDL-C, HDL-C for analysis. Eventually, we found that postoperative HDL-C could act as an independent prognostic marker in GC among all lipid molecules. Although the underlying mechanism is unclear, stomach as one of the most important organs of the digestive system, patients will experience changes in dietary habits, poorer nutritional status, weight loss, and lower serum lipids after surgery, which requires further investigation.

It should be noted that our study has several potential limitations. First, it is a retrospective, single-center study, so the representativeness of patients is less than those in a prospective and multi-center study, and the results may be biased. Second, the results of this study are necessary to further validate by a mechanistic detail *in vivo* and *in vitro*. In spite of these limitations in this study, our analysis firstly tried to elucidate the impact of postoperative serum lipids on the prognosis in gastric cancer patients.

## Conclusions

This study suggests that low serum HDL-C level of 6 months after operation is associated with poor prognosis in gastric cancer. Thus, we recommend measurement of serum HDL-C level after 6 months of gastrectomy to predict the prognosis of gastric cancer.

## Data availability statement

The original contributions presented in the study are included in the article/supplementary material. Further inquiries can be directed to the corresponding authors.

## Ethics Statement

The studies involving human participants were reviewed and approved by the ethics committee of the Chinese PLA General Hospital. The patients/participants provided their written informed consent to participate in this study.

## Author contributions

CL, YF, QL, and XY have contributed equally to this work and share first authorship. LP and ND have contributed equally to this work and share last authorship. All authors contributed to the article and approved the submitted version.

## Conflict of Interest

The authors declare that the research was conducted in the absence of any commercial or financial relationships that could be construed as a potential conflict of interest.

## Publisher’s note

All claims expressed in this article are solely those of the authors and do not necessarily represent those of their affiliated organizations, or those of the publisher, the editors and the reviewers. Any product that may be evaluated in this article, or claim that may be made by its manufacturer, is not guaranteed or endorsed by the publisher.
